# Big brown bats (*Eptesicus fuscus*) successfully navigate through clutter after exposure to intense band-limited sound

**DOI:** 10.1038/s41598-018-31872-x

**Published:** 2018-09-10

**Authors:** Andrea Megela Simmons, Alexandra Ertman, Kelsey N. Hom, James A. Simmons

**Affiliations:** 1 Department of Cognitive, Linguistic, and Psychological Sciences, Brown University, Providence, RI 02912 USA; 2 Department of Neuroscience, Brown University, Providence, RI 02912 USA; 3 Present Address: Department of Biology, The Graduate Center, City University of New York, 365 5th Avenue, New York, NY 10016 USA

## Abstract

Echolocating big brown bats fly, orient, forage, and roost in cluttered acoustic environments in which aggregate sound pressure levels can be as intense as 100 to 140 dB SPL, levels that would impair auditory perception in other terrestrial mammals. We showed previously that bats exposed to intense wide-band sound (116 dB SPL) can navigate successfully through dense acoustic clutter. Here, we extend these results by quantifying performance of bats navigating through a cluttered scene after exposure to intense band-limited sounds (bandwidths 5–25 kHz, 123 dB SPL). Behavioral performance was not significantly affected by prior sound exposure, with the exception of one bat after exposure to one sound. Even in this outlying case, performance recovered rapidly, by 10 min post-exposure. Temporal patterning of biosonar emissions during successful flights showed that bats maintained their individual strategies for navigating through the cluttered scene before and after exposures. In unsuccessful flights, interpulse intervals were skewed towards shorter values, suggesting a shift in strategy for solving the task rather than a hearing impairment. Results confirm previous findings that big brown bats are not as susceptible to noise-induced perceptual impairments as are other terrestrial mammals exposed to sounds of similar intensity and bandwidth.

## Introduction

In a number of vertebrate species, exposure to intense sound produces subsequent temporary (temporary threshold shift, TTS) hearing losses, in which thresholds to test sounds presented up to several hrs post-exposure can be elevated by as much as 40 dB. The severity and duration of these auditory impairments depend on the acoustic features (bandwidth, duration) of the prior exposure as well as on the duration of the post-exposure recovery time^1,2^. Previously, we reported^3,4^ that echolocating big brown bats (*Eptesicus fuscus*) may be less susceptible to TTS than expected from psychophysical experiments in other terrestrial mammals. In one experiment, we exposed seven big brown bats to wideband sound covering their audiometric range^5^ (10–100 kHz) for 1 hr at sound levels [116 dB sound pressure level (SPL) *re* 20 μPa] within the range they experience in the natural environment (100–140 dB SPL)^6^. Bats’ thresholds to two-harmonic frequency modulated (FM) sweeps similar to their natural biosonar pulses did not increase significantly either 20 min or 24 hr post-exposure^3^. In further work, we exposed six bats for 1 hr to intense band-limited sounds (bandwidths 10–50 kHz, mean level 116 dB SPL)^4^. When assessed at recovery times of 2 and 5 min after exposure, their thresholds to single harmonic FM sweeps (FM1; downward sweeping from 50 to 25 kHz^7^) increased by only 2 dB from those estimated at pre-exposure or in sham exposure conditions. In contrast, other terrestrial mammals can experience significant TTS (8–40 dB) lasting up to several hrs or even days after exposure to similarly-intense band-limited sounds within their audiometric ranges^1,8–11^. A criterion of a minimum threshold increase of 6 dB has been adopted to define a significant TTS^2,12^. By this criterion, big brown bats do not experience TTS, although an exhaustive test of stimulus parameters that may affect hearing thresholds after intense sound exposure has not been carried out in any species of echolocating bat.

Not only is the psychophysical performance of big brown bats unaffected by previous exposure to intense wideband sound, but also their ability to navigate by biosonar through an acoustically-challenging environment is unimpaired. Hom *et al*.^13^ trained four big brown bats to fly down a narrow, curved corridor through a dense array of plastic hanging chains providing strong clutter echoes^14,15^ similar to the complex acoustic reflectors bats encounter while navigating through vegetation^16^. Bats navigated the corridor accurately, with few errors, both before and 20 min after exposure to 1 hr of intense wideband sound (10–100 kHz, 116 dB SPL). Moreover, the characteristics (number of pulses; interpulse intervals, IPI) of their biosonar broadcasts remained stable before and after sound exposure. Bats emit pulses either singly or in IPI patterns described as sonar sound (strobe) groups^14,17–20^, in which 2–4 pulses (doublets, triplets, quadruplets) separated by short IPIs alternate with pulses separated by long IPIs. The alternation of IPIs from short-to-long allows bats to distinguish echoes from close obstacles (in this case, rows of chains producing echoes at short delays) while simultaneously planning a path to the goal (the back wall, producing echoes at longer delays), thus reducing pulse-echo ambiguity^14^. In difficult experimental tasks, bats alter the temporal patterning of IPIs by producing more complex (doublet, triplet, quadruplet) sonar sound groups, thus changing the distributions of short and long IPIs^14,15,19^. A change in temporal patterning after sound exposure towards more complex sonar sound groups and more short IPIs would suggest that the experimental task became perceptually more difficult after the exposure. In the experiment by Hom *et al*.^13^, neither the complexity of sonar sound groups nor the distributions of short and long IPIs varied before and after sound exposure. These data indicate that prior intense wideband sound neither impaired the ability to navigate by biosonar nor increased the perceptual difficulty of the task.

It is possible that the experimental parameters tested by Hom *et al*.^13^ were not sufficiently sensitive to uncover any post-exposure impairments in behavioral performance or alterations in the temporal patterning of biosonar pulses. Indeed, many psychophysical studies of TTS in other terrestrial mammals have quantified decreases in hearing sensitivity after band-limited, rather than wideband, exposures and at shorter post-recovery times (2–4 min rather than 20 min^1,2,8^). To determine whether the results of Hom *et al*.^13^ could be replicated with different experimental parameters, we exposed bats to sounds of various bandwidths (BW) at the same duration (1 hr) as used in that experiment, but we increased the level of exposure to a mean level of 123 dB SPL and we assessed bats’ performance in navigating a denser chain array beginning at 2 min post-exposure. These exposure parameters are more comparable to those used in TTS experiments with other terrestrial vertebrates^1,2,8–10^. In addition, we now include a sham (no sound) exposure condition to assess how performance might be affected by repeated testing. We predicted that bats’ performance, as indexed by numbers of errors and temporal patterning of biosonar pulses, would remain stable after sound and after sham exposures, suggesting that they retained their hearing sensitivity and their ability to perform a complex biosonar task.

## Results

**Figure 1:**
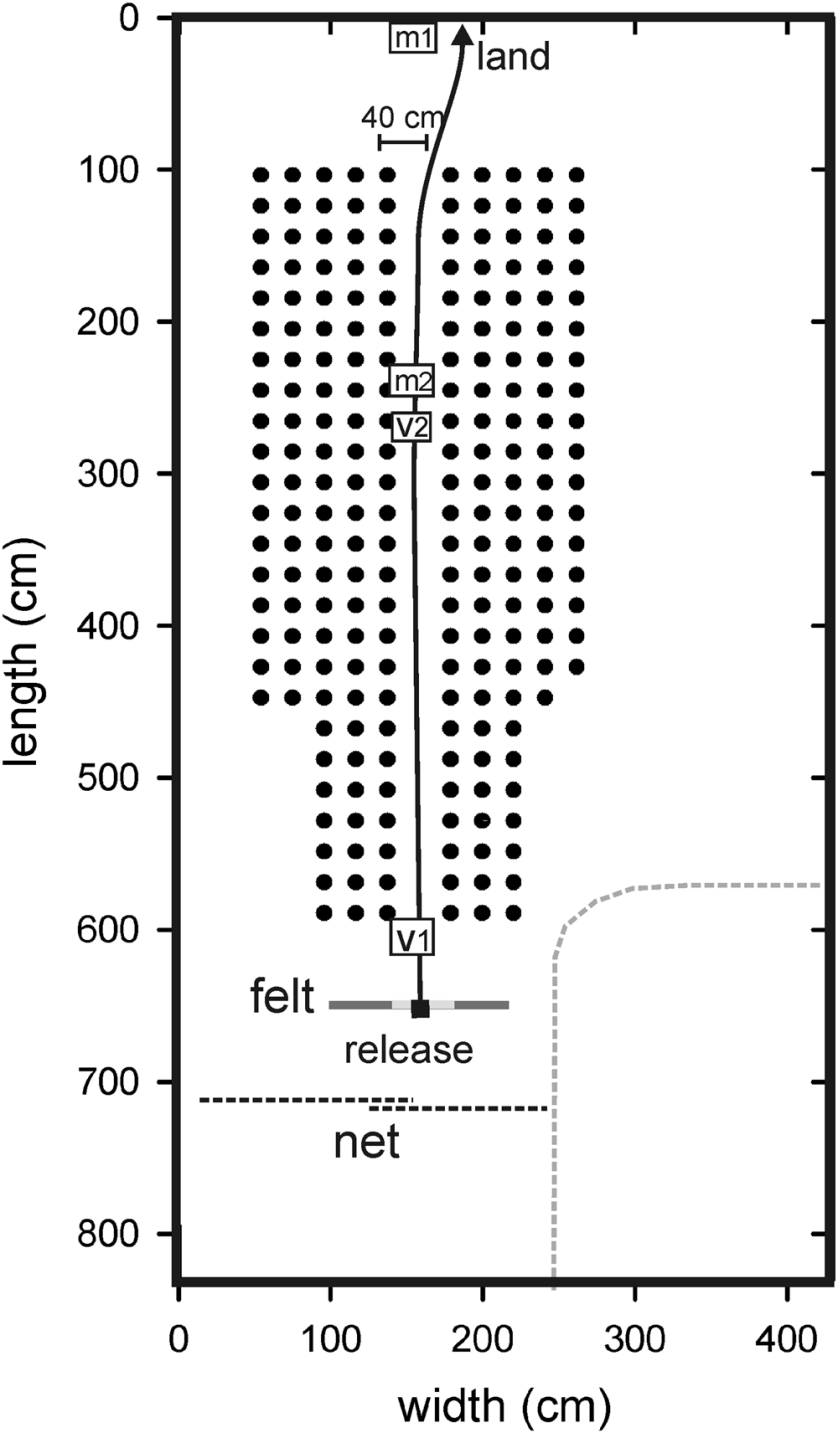
Plan view of the flight room. Closed circles show the positions of the hanging chains, arranged in rows and columns separated by 20 cm. The 40-cm-wide corridor through the chains and a typical flight trajectory are shown by the open space and the solid line. Bats were launched at the square labeled “release”. The thick gray line in front of the release point shows the position of the hanging felt. The bat had to fly through a hole in this felt to reach the entrance to the corridor. The dashed black lines behind the release point show the position of a hanging net that prevented the bat from flying outside the boundaries of the chain array. The landing position on the back wall is indicated (“land”). The locations of the two microphones are shown by the open squares labeled m1 and m2, and the position of the video monitors are shown by the open squares labeled v1 and v2. The dashed gray lines to the bottom right of the figure show the location of acoustic foam separating the chain array from equipment used for other experiments.

**Figure 2:**
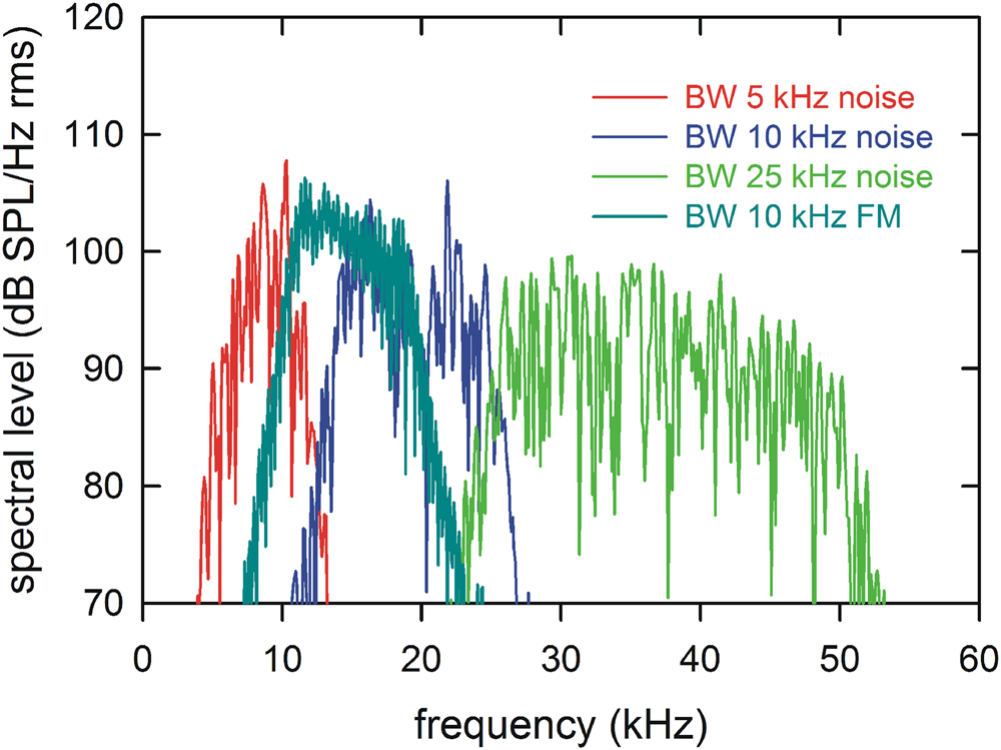
Spectral levels of the four sounds used for exposures. Values are plotted on a vertical scale set by a calibration reference tone of 20 kHz at 120 dB SPL rms. Spectral level is given as dB SPL/Hz rms from spectra computed over 2048 sample segments to keep all values comparable. Level is slightly higher for the BW 10 kHz FM compared to BW 5 kHz and BW 10 kHz. All sounds were presented at a mean level of 123 dB SPL rms (variation 119–124 dB SPL at different locations in the holding cage used for exposures).

The ability of four bats to navigate a corridor through a chain array (Fig. 1) was assessed before and after exposure to 1 hr of band-limited sound [exposure test days E1 (24 hr pre-exposure), E2 (2 min post-exposure), and E3 (24 hr post-exposure)], and before and after sham (no sound) exposure [sham test days S1 (24 hr pre-sham), S2 (2 min post-sham), and S3 (24 hr post-sham); see Table 1; spectral levels of exposure sounds are shown in Fig. 2]. If bats experienced any perceptual impairment due to exposure, then this should be reflected in increased errors on test day E2 (2 min post-exposure) compared to both test day E1 (24 hr pre-exposure) and test day S2 (2 min post-sham). Moreover, because bats typically produce more pulses with shorter IPIs in challenging tasks^14,15,17–20^, we expected pulse numbers to increase, IPIs to decrease, and the temporal patterning of IPIs to alter after sound exposure but not after sham exposure.

**Table 1:** Behavioral performance on each test day by each bat.

Stimulus BW (range)	Bat	Order^a^	Test Day^b^	Successful flights^c^	Unsuccessful flights^d^	Bayesian OR^e^ (95% CI)
1: BW 25 kHz (25-50 kHz)	A	1	E1	12	0	
			E2	10	2	5.96 (0.30, 11.679)^f^
			E3	12	0	
			S1	12	1	
			S2	12	0	0.17 (0.01, 3.28)^g^
			S3	12	0	
2: BW 10 kHz (15-25 kHz)	B	1	E1	12	1	
			E2	11	7	5.11 (0.82, 31.94)^f^
			E3	10	4	
			S1	12	0	
			S2	10	2	0.38 (0.08, 1.87)^g^
			S3	11	1	
	C	2	E1	12	0	
			E2	12	0	1.00 (0.01, 83.92)^f^
			E3	12	0	
			S1	12	0	
			S2	12	0	1.00 (0.01, 83.92)^g^
			S3	12	0	
3: BW 10 kHz FM	B	2	E1	10	3	
			E2	8^i^	14	4.74 (1.14, 19.67)^f,h^
			E3	12	2	
			S1	12	1	
			S2	12	0	0.02 (0.001, 0.40)^g,h^
			S3	11	1	
	C	1	E1	12	1	
			E2	10	2	1.84 (0.23, 14.63)^f^
			E3	11	1	
			S1	12	0	
			S2	12	1	0.54 (0.07, 4.30)^g^
			S3	12	0	
4: BW 5 kHz (5-10 kHz)	B	2	E1	12	1	
			E2	12	2	1.62 (0.21, 12.74)^f^
			E3	12	2	
			S1	12	0	
			S2	12	0	0.19 (0.01, 3.74)^g^
			S3	12	0	
	D	1	E1	12	0	
			E2	12	6	14.83 (0.80, 274.11)^f^
			E3	12	0	
			S1	10	2	
			S2	11	2	0.44 (0.09, 2.14)^g^
			S3	12	0	

^a^Order of condition: 1 = sound exposure first; 2 = sham exposure first.

^b^E1: 24 hr pre-exposure; E2: 2 min post-exposure; E3: 24 hr post-exposure; S1: 24 hr pre-sham; S2: 2 min post-sham; S3: 24 hr post-sham.

^c^Each bat was flown for a maximum of 10–12 successful, rewarded flights on each test day.

^d^Unsuccessful flights include failures to launch, colliding with chains, or landing on the floor within the corridor.

^e^Bayesian odds-ratio (OR) with confidence interval (CI). CIs that include the number 1 are not significant.

^f^Comparing between E2 and E1.

^g^Comparing between E2 and S2.

^h^Statistically-different comparison.

^i^This bat could be flown only for a maximum of 8 successful flights because of depletion of the reward allotment.

### Bats continue to navigate the chain array successfully after sound exposure

Table 1 shows the numbers of successful and unsuccessful (error) flights for each bat in the exposure (test days E1, E2, E3) and in sham (test days S1, S2, S3) conditions. We compared the numbers of successful and unsuccessful flights between test days E2 and E1 and between test days E2 and S2 for each bat and exposure sound using Bayesian odds-ratios (Table 1). These comparisons show that, even though numbers of errors on test day E2 (1–14 errors, with median of 2) were higher than on test days E1 (median of 1) or S2 (median of 1), bats largely retained their ability to navigate the corridor successfully after sound exposure. Only one bat (Bat B) made significantly more errors after exposure to one sound (stimulus 3, BW 10 kHz FM) compared to his performance at 24 hr pre-exposure or 2 min post-sham exposure. The other bat, Bat C, who was also exposed to stimulus 3 maintained good performance, suggesting that individual differences between bats overshadowed any effects of prior sound exposure.

**Figure 3:**
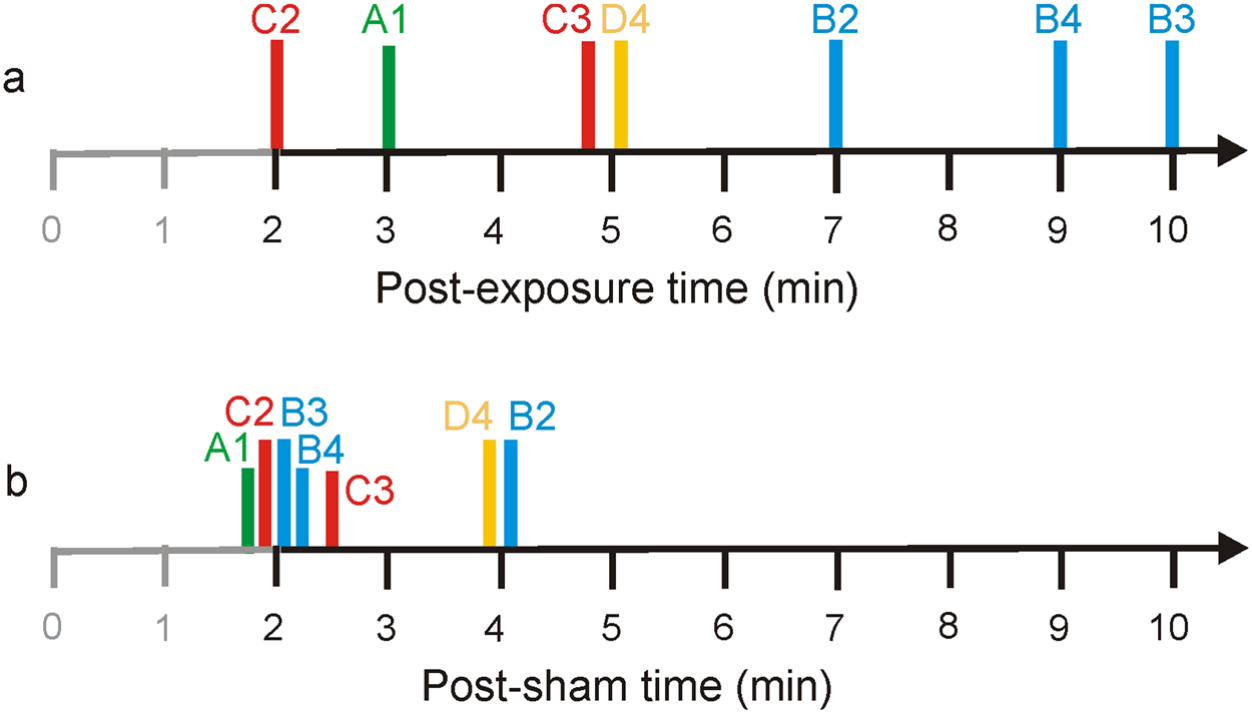
Time to maintained successful flights for each bat and each exposure sound. (**a**) Test day E2, 2 min post-exposure. Lines are offset around the 5 min mark to avoid overlap. (**b**) Test day S2, 2 min post-sham. Lines are offset around the 2 min mark to avoid overlap. Each bat is identified by a unique color and letter: Bat A = green; Bat B = blue; Bat C = red; and Bat D = yellow. Exposure sounds are identified by numbers: 1 = BW 25 kHz; 2 = BW 10 kHz; 3 = BW 10 kHz FM; 4 = BW 5 kHz. Unsuccessful flights (errors) were typically concentrated within the first 4 min post-exposure and post-sham. After exposure to stimulus 3, Bat C made one error at 3 min and one error at 5 min. After exposure to stimulus 4, Bat B made one error at 5 min and one error at 9 min. All errors resolved by 10 min post-exposure.

The time-course of performance for all bats on test day E2 was examined to determine at what point in the flight series, if errors occurred, successful flights resumed without any additional errors (regardless of the total number of errors). For most bats, errors were clustered early in the flight series on test day E2, from 2 min to 5 min post-exposure, with a median of 5 min (Fig. 3a). The outlier was Bat B, who resumed and maintained successful flights 7 min after exposure to stimulus 2, 10 min after exposure to stimulus 3, and 9 min after exposure to stimulus 4 (the two errors to this stimulus occurred at 4 and 9 min post-exposure). Any unsuccessful flights that occurred on test day S2 also occurred early in the flight series, between 2 and 4 min post-sham exposure, with a median of 2 min (Fig. 3b). A Wilcoxon signed-rank test shows that the differences in time to resume successful flights on test days E2 and S2 are statistically significant (*P* = 0.027), due to the data from Bat B. Even when including those values, it is clear that any negative impact of sound exposure on performance in the experimental task dissipated rapidly.

### Biosonar pulse emission in successful flights is stable after sound exposure

**Figure 4:**
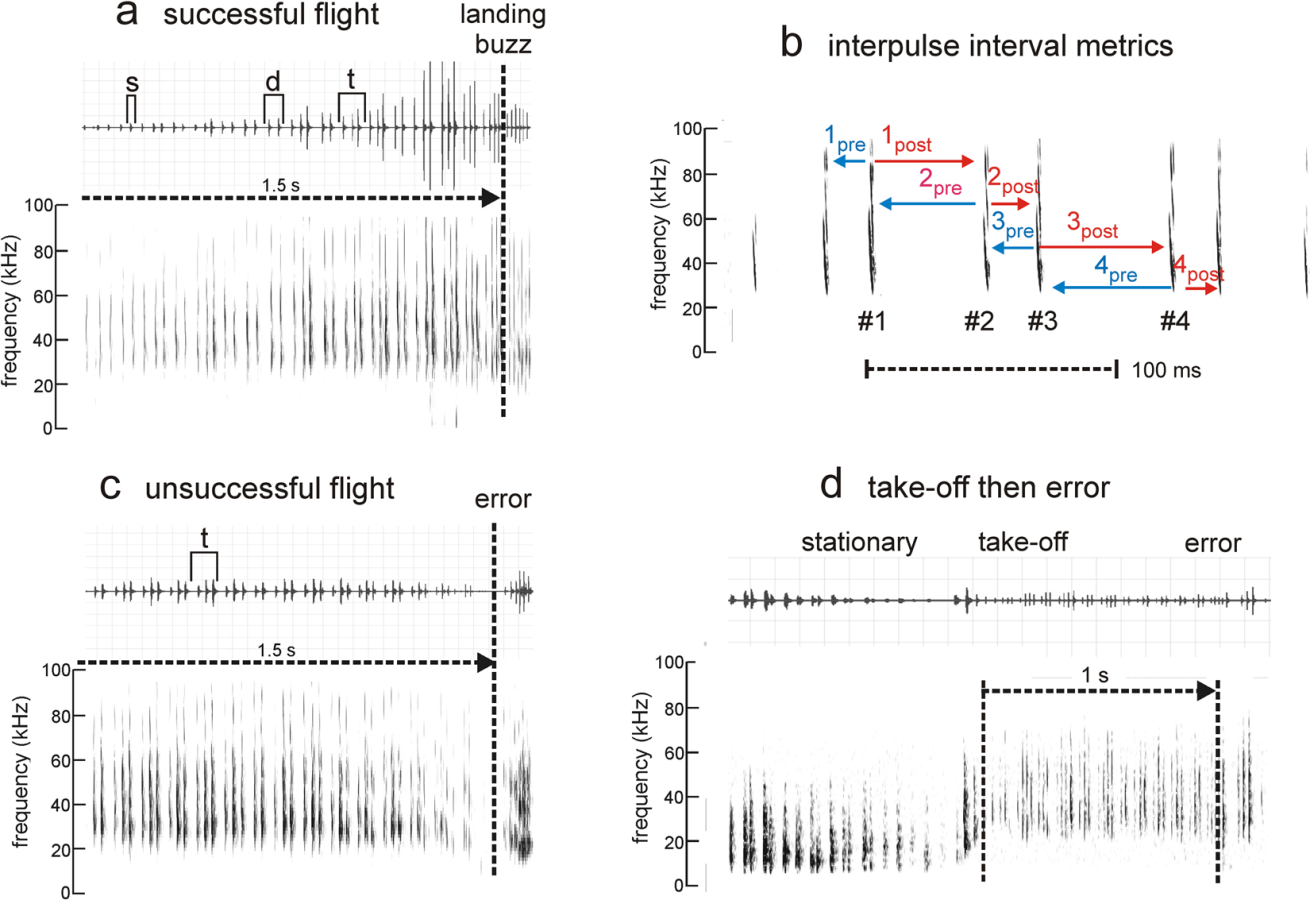
Pulse emission sequences in successful and unsuccessful flights. All sequences are from Bat B recorded on E2, 2 min post-exposure to BW 10 kHz FM. Plots (**a,c,d**) show time waveforms (top) and spectrograms (bottom) for the bat’s own broadcasts as recorded by the wall microphone. (**a**) Series of FM broadcasts emitted during a successful flight terminating in a landing on the wall. The dashed vertical line shows the demarcation of the flight from the landing buzz. A time segment of 1.5 s prior to the landing buzz was used to calculate pulse number and IPIs. The time waveform plot shows the presence of sonar sound groups: s-single; d = doublet; t = triplet. (**b**) Visualization of metrics used to calculate IPI and sort by pre-IPIs (blue intervals) and post-IPIs (red intervals) for four sample pulse intervals. This is necessary because mean IPI values for flights do not describe sound emission patterns. (**c**) Series of FM broadcasts emitted by the bat during a flight terminating in an error. In this flight, the bat struck and then landed on a chain near the end of the corridor instead of on the back wall. (**d**) Series of FM broadcasts emitted by the bat while stationary prior to take-off, during take-off, and then during the flight terminating in an error (time in corridor demarcated by the two dashed vertical lines). In this case, the bat landed on the floor of the corridor.

An example of the sequence of emitted pulses from one bat (Bat B) on a successful flight on test day E2 is illustrated in Fig. 4a. For analysis, flights were truncated to a standard duration of 1.5 s (range of 1.43–1.55 s), representing the duration of sustained flight within the corridor after the bat entered and before the onset of the landing buzz. Choosing a standard duration of flight allowed us to compare performance between bats with different flight speeds. Within this 1.5 s interval, numbers of pulses, IPIs, and post-IPI and pre-IPI intervals (Fig. 4b) were quantified. Because all four bats were not exposed to the same stimulus sound and only two of the four were exposed to more than one sound, we performed GLM repeated measures analysis of variance (RM-ANOVA) on number of pulses and IPIs for each sound exposure stimulus separately, with Test Day as the repeated measure and Bat as the between-subjects variable when appropriate. In order to compare a standard number of flights across bats, data from the first 10 successful flights (out of a maximum of 12 flights, see Table 1) on each test day were included in these analyses.

**Figure 5:**
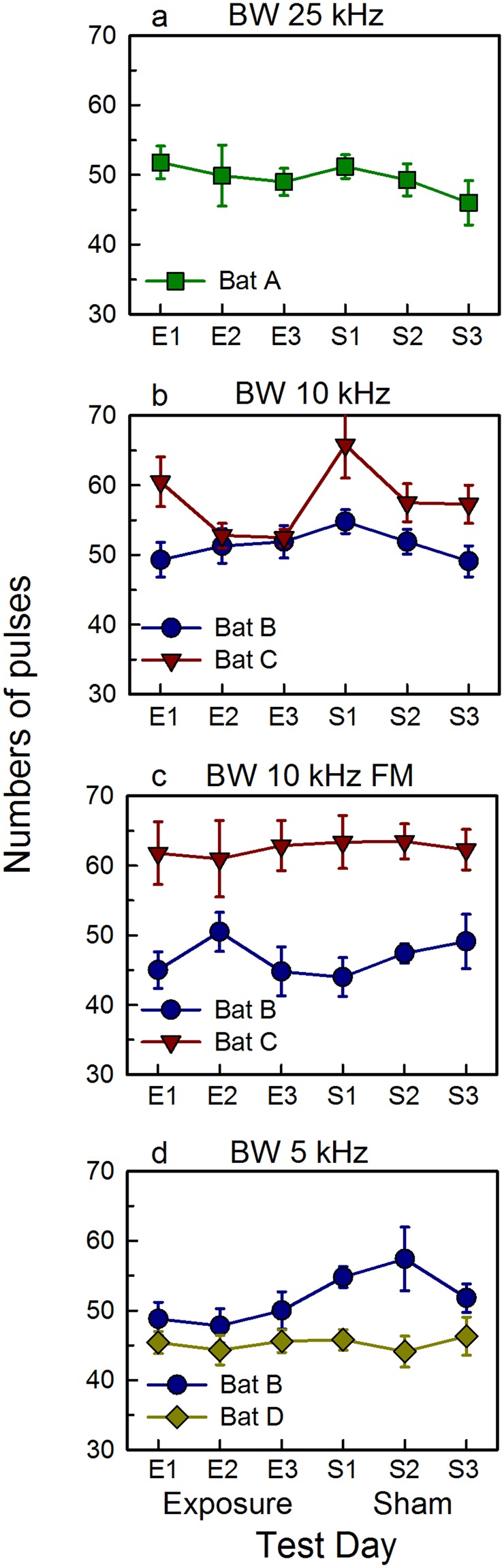
Numbers of pulses emitted during the flight task vary across test days and between individual bats, but do not significantly increase after sound exposure. Data are shown for each exposure sound separately. (**a**) BW 25 kHz; (**b**) BW 10 kHz; (**c**) BW 10 kHz FM; (**d**) BW 5 kHz. In each plot, data points are means +/−  1 standard deviation in the first 10 successful flights on each test day (x axis), with individual bats identified by a unique color and symbol. Test days are separated into the two conditions of Exposure and Sham. Test day E1 is the 24 hr pre-exposure day; test day E2 is the 2 min post-exposure day; test day E3 is the 24 hr post-exposure day; test day S1 is the 24 hr pre-sham day; test day S2 is the 2 min post-sham day; and test day S3 is the 24 hr post-sham day. Although the Exposure condition is plotted first, the order in which these two conditions was presented varied (see Table 1). Over the entire dataset, there is no statistical increase in number of emitted pulses on test day E2 compared to test days E1 or S2, as would be predicted by a hypothesis of perceptual impairment.

For Bat A, exposed to stimulus 1 (BW 25 kHz), numbers of pulses varied significantly across the six test days (F_2.562,23.059_ = 6.029, *P* = 0.005; Fig. 5a). Numbers of pulses decreased over the three days in the exposure condition, increased on test day S1, and then decreased again over the three days in the sham condition. Bonferroni-adjusted pairwise comparisons (critical value *P* = 0.025) showed no significant differences between the numbers of pulses between test days E1 and E2, nor between test days E2 and S2. Mean IPI (Supplemental Fig. 1a) also varied across the six test days (F_4.705,1919.765_ = 4.654, *P* < 0.0001). IPI increased significantly (*P* < 0.025) over the three days in the sham condition but was stable over the three test days in the exposure condition. These data show that pulse number and IPI can vary over repeated testing but not in a manner directly related to sound exposure.

The two bats (Bats B and C) exposed to stimulus 2 (BW 10 kHz) emitted significantly different numbers of pulses during flights (F_1,18_ = 132.97, *P* < 0.0001; Fig. 5b). Numbers of pulses varied significantly across the six test days (F_5,90_ = 28.579, *P* < 0.0001). Pairwise comparisons show statistical differences (*P* = 0.014) between test days E1 and E2, driven by a decline in numbers of pulses on E2 by Bat C. This decrease in pulse number persisted on test day E3. Numbers of pulses were larger on test day S2 compared to E2 (*P* = 0.022), again driven by data from Bat C. Neither of these effects would be predicted by a hypothesis of exposure-induced auditory impairment. Pulses from these two bats differed significantly in IPI (F_1,952_ = 236, *P* < 0.0001), and IPI differed significantly across the six test days (F_4.88,4650.6_ = 24.538, *P* < 0.0001; Supplemental Fig. 1b), but were not consistently shorter on E2 compared to E1 or S2.

The two bats (Bats B and C) exposed to stimulus 3 (BW 10 kHz FM) differed significantly in numbers of emitted pulses (F_1,14_ = 304.9, *P* < 0.0001), but numbers of pulses did not differ significantly over the six test days (*P* = 0.121; Fig. 5c). Bats emitted pulses differing in IPI (F_1,968_ = 794.2, *P* < 0.0001), and IPI varied across the six test days (F_4.91, 4752.6_ = 6.475, *P* < 0.0001; Supplemental Fig. 1c). IPI differed significantly between test days E1 and E2 (*P* < 0.0001), driven by a decrease in IPI on E2 by Bat B. This bat made significantly more flight errors on test day E2 (Table 1), and the shortening of IPI during subsequent successful flights is consistent with a hypothesis of perceptual impairment. On the other hand, IPIs were stable on test days E2 and S2 for this bat, suggesting that the change in IPI may not be a specific consequence of prior sound exposure.

The two bats (Bats B and D) exposed to stimulus 4 (BW 5 kHz) emitted significantly different numbers of pulses (F_1,18_ = 208.8, *P* < 0.0001; Fig. 5d). Numbers of pulses varied significantly across the six test days (F_3.38, 60.866_ = 11.708, *P* < 0.0001), but remained stable on test days E1 and E2. Although both of these bats made some flight errors on test day E2 (Table 1), neither increased pulse number on subsequent successful flights. Numbers of pulses were greater on test day S2 than on E2 (*P* < 0.0001), driven by the data from Bat B. Bats differed significantly in IPI (F_1,856_ = 316.2, *P* < 0.0001) and IPI differed significantly across the six test days (F_4.931, 4220.66_ = 8.296, *P* < 0.0001; Supplemental Fig. 1d). IPIs decreased on test day S2 compared to E2 (*P* < 0.001), driven by the data from Bat B, which is not in the direction predicted by a hypothesis of perceptual impairment.

**Figure 6:**
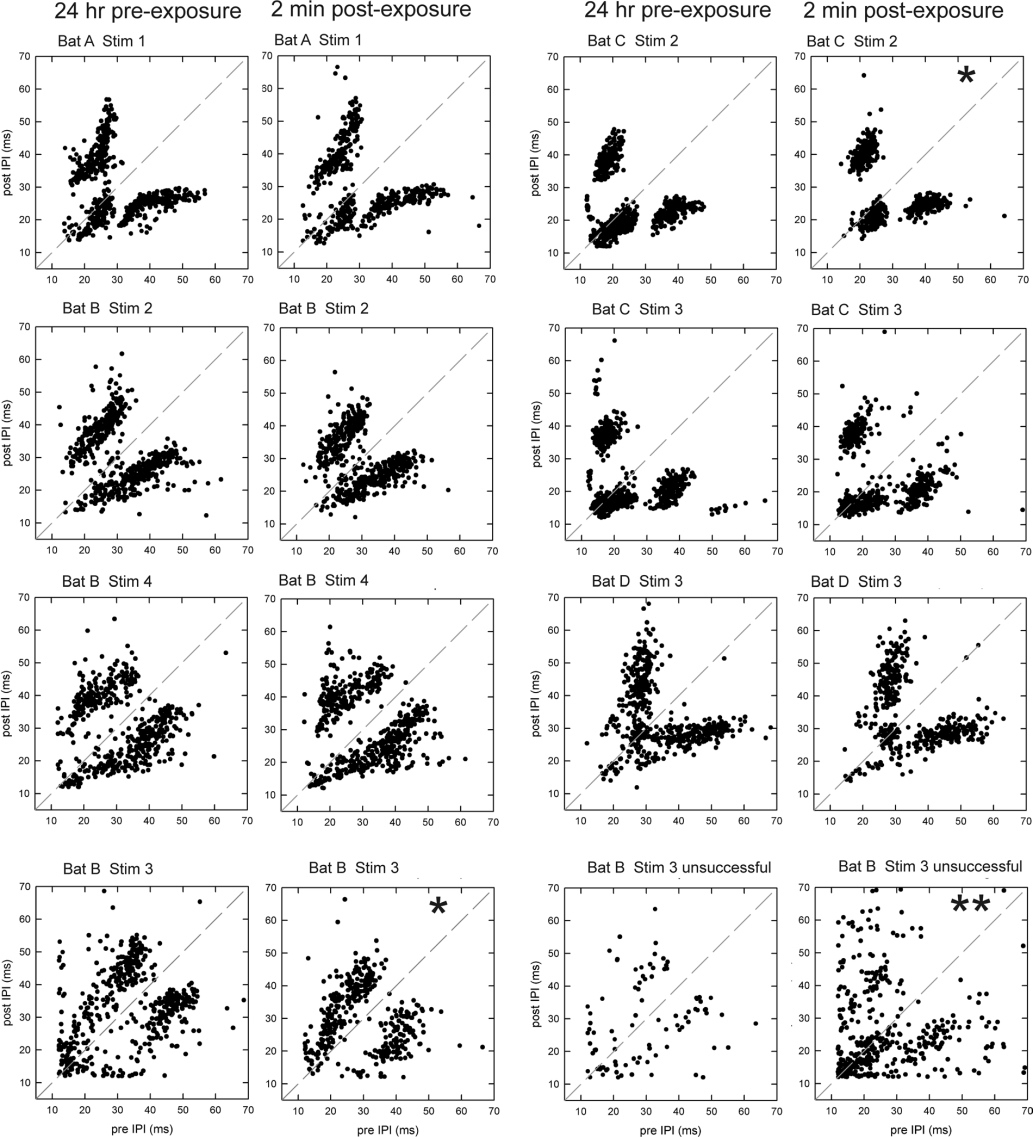
Clustering of IPIs varies between individual bats. Each plot shows the post-IPI (y axis) plotted against the pre-IPI (x axis; see Fig. 4b) for each individual bat on the 24 hr pre-exposure (test day E1) and the 2 min post-exposure (test day E2) test days. Stim = sound stimulus used for exposure. Data are from successful flights, except in the two bottom right plots for Bat 3 stim 3 where data from unsuccessful flights are shown. The dashed diagonal line in each plot shows the expected distribution of data points if IPIs did not alternate between short and long (that is, were produced as singlets and not clustered into sonar sound groups). The distribution of IPI values differs between bats, showing that each bat adopted a unique strategy for navigating the corridor. The single asterisks (Bat B stim 3 successful flights; Bat C, stim 2) show the presence of significant differences between the 24 hr pre-exposure and the 2 min post-exposure condition. The double asterisks (Bat B, stim 3 unsuccessful flights) show a significant difference in IPI distributions between successful and unsuccessful flights at 2 min post-exposure. Bat B made more triplets in unsuccessful than in successful flights, as shown by the increased clustering at short IPI values, while also producing more long IPIs.

To assess whether the temporal patterning of IPIs changed after sound exposure, we examined the distribution of post-IPI and pre-IPI intervals (Fig. 4b) on successful flights on each test day for each bat. Graphs of the distributions of these intervals on test days E1 (24 hr pre-exposure) and E2 (2 min post-exposure) are shown in Fig. 6. These graphs show clusters of short and long IPIs; clusters of short IPIs indicate the presence of complex sonar sound groups (Fig. 4a). In successful flights, bats emitted singlets, doublets and triplets, but in different patterns (distributions), suggesting that each bat adopted a unique strategy to solve the task. The Kolmogorov-Smirnov test was used to assess statistical similarity of IPI distributions between all test days in the exposure and sham conditions. For most comparisons, *P* values were greater than the Bonferroni-adjusted value of *P* = 0.01. Two significant trends did emerge–Bat B after exposure to stimulus 3 and Bat C after exposure to stimulus 2. Bat B emitted more short IPIs on test day E2 compared to E1, while Bat C emitted more short IPIs on test day E1 compared to E2. These differences suggest that the task became perceptually more difficult to Bat B but not to Bat C after exposure, again highlighting individual differences between bats in performance. These data also show that the distributions of IPIs vary with experience with the task, regardless of any experimental manipulation.

It is possible that, if the bats experience any temporary hearing loss as a result of the prior sound exposure, they would then emit pulses at higher amplitudes so as to receive higher amplitude echoes. Because the microphone was on the back wall, it acquired pulse amplitudes that increased as the bat flew down the corridor (Fig. 4a). We analyzed changes in pulse amplitude on successful flights, as measured by the voltage on the wall microphone uncorrected for any change in head or beam aims, over all test days using RM-ANOVA. Pulse amplitudes varied between bats and across test days, but within the entire dataset there was no significant increase in amplitudes specifically on test day E2 compared to test days E1 or S2. We then analyzed separately data from the two bats both exposed to stimulus 3. These two bats performed differently after exposure, with Bat B making significantly more errors (14) and Bat C showing stable performance (only two errors, not significant). Bat C emitted pulses with higher amplitude than Bat B on all test days (F_1,1004_ = 2486, *P* < 0.0001), and with amplitudes larger on sham test days than on exposure test days. Bat B significantly increased pulse amplitude on both test days E2 and E3 over that on E1 (*P* < 0.0001), rather than on E2 alone. These data show that pulse amplitude, as measured in this experiment, varies between individual bats and across experimental days, but does not increase specifically on successful flights 2 min post-exposure.

### Temporal patterning of biosonar pulses changes during unsuccessful flights

Examples of the sequence of pulse emission on two unsuccessful flights on test day E2 (data from Bat B) are shown in Fig. 4c,d. Figure 4c shows an example of an unsuccessful flight in which pulses were emitted largely in triplets, a pattern that differed from that shown in the successful flight from the same bat in Fig. 4a, which shows singlets, doublets, and triplets. On this unsuccessful flight, pulse amplitude decreased rather than increased as the bat flew down the corridor, and the bat landed on a chain in the corridor rather than on the back wall. Figure 4d shows an example of another unsuccessful flight in which this bat did not launch immediately from the experimenter’s hand (as is typical on successful flights), but instead remained there while emitting high amplitude, low spectral frequency pulses characteristic of communication signals^21^. Once launched, pulse spectral frequency increased to that typical of biosonar signals. Durations of unsuccessful flights (exclusive of failures to launch) from the time the bat entered the corridor ranged from 0.167 s, reflecting an error near the entrance, to 1.52 s, reflecting an error near the exit of the corridor. We analyzed the distribution of IPIs on test day E2 on all of Bat B’s successful and unsuccessful (excluding those in which he failed to launch) flights across all sound exposure stimuli. Kolmogorov-Smirnov tests confirmed that IPI distributions differed statistically (*P* = 0.011), with more short IPIs in unsuccessful than in successful flights (Fig. 6).

We then analyzed pulse amplitudes on unsuccessful flights to determine whether bats emitted pulses at higher amplitudes on these flights in an attempt to receive higher amplitude echoes, as expected if they experienced a loss of hearing sensitivity. We compared amplitudes of Bat B’s pulses in time-matched segments of 8 successful and 8 unsuccessful flights after exposure to stimulus 3. Time-matched segments were used because unsuccessful flights were typically shorter than successful flights, since errors always occurred before the bat exited the corridor to reach the back wall. Results of paired t-tests showed that pulse amplitudes were significantly lower in unsuccessful flights [*t* (285) = −7.21, *P* < 0.0001], an effect contrary to a hypothesis of perceptual impairment.

## Discussion

Echolocating big brown bats fly, orient, forage, and roost in cluttered acoustic environments where they are exposed to their own biosonar pulses, to the pulses of other bats, and to echoes from multiple surrounding objects. Aggregate sound pressure levels in these environments can be as intense as 100 to 140 dB SPL, levels that would impair auditory perception in other terrestrial mammals. The results of this experiment show that big brown bats are able to navigate successfully through dense acoustic clutter after exposure to band-limited sound within this intensity range. The particular exposure conditions used in this study produced neither consistent nor long-lasting perceptual impairments; individual variability between bats was a dominant feature of the results. Even though some bats made more unsuccessful flights after sound than after sham exposures, numbers of these errors were statistically larger only in one outlying condition. Thus, by a Bayesian analysis, performance is invariant to the experimental manipulation, the sound exposure^22^. Unsuccessful flights, when they occurred, were concentrated at the start of the flight series, beginning at 2 min post-exposure but resolving quickly, by 10 min post-exposure. Performance 24 hr later was consistent with that at pre-exposure and on sham test days, indicating an absence of long-term residual effects of exposure on performance. These new findings are consistent with our earlier work showing that big brown bats are not as susceptible to auditory impairments following intense sound exposure as would be expected from research in other terrestrial mammals.

Four sounds with different BWs and spectral levels were used for exposure, but all sound exposure stimuli were presented at the same mean level and produced similar results. Exposure to stimulus 3, with the highest peak spectral level and of a frequency range that could have produced effects analogous to the half-octave shift described for other terrestrial mammals^23^, impaired performance in one bat but not in a second bat. It is possible that this particular stimulus was at the border of an effect/no effect, likely due to its somewhat higher spectral level. FM sweeps of similar frequency content and BW presented at somewhat lower spectral levels do not impair psychophysical performance^4^. Future experiments testing the impact of sound exposure stimuli at even higher spectral levels will be important in delineating the limits of the bat’s lessened susceptibility to TTS.

The pattern of results obtained here is consistent with our previous findings exploring the impact of prior sound exposure on psychophysical hearing thresholds^3,4^ and flight performance^13^. Simmons *et al*.^4^ observed only small (mean of 2 dB) increases in hearing thresholds of big brown bats tested 2 or 5 min after exposure to band-limited sound (BW 10–40 kHz) at lower levels (mean of 116 dB SPL). A threshold increase of 2 dB is below the criterion for TTS^2,12^. Our data show that behavioral performance when challenged with strong acoustic clutter is not only unaffected when assessed 20 min post-exposure to wide-band noise^13^, but also is largely maintained when exposed to BW 5–25 kHz sounds at even higher levels. The small numbers of unsuccessful flights and the rapid recovery time would not be predicted from experiments on TTS in other terrestrial mammals, where similar durations and levels of sound exposures produce increases in behavioral thresholds that can last hours^1,8–11^. Ryan and Bone^10^ exposed guinea pigs to two-octave band noise at 100–120 dB SPL for 1 hr and observed threshold increases of 30–40 dB at a recovery time of 30 min. Longenecker *et al*.^11^ exposed mice, mammals that also hear within the ultrasonic range, to band-limited noise (8–17 kHz) for 1 hr at 116 dB SPL. Behavioral thresholds measured in a prepulse inhibition task were “dramatically increased” immediately after exposure and remained significantly elevated (by 10–30 dB in individual animals) at 24 hr and 3 months post-exposure. If big brown bats had similar susceptibility to these other species, then the exposure parameters used in our experiments would have produced a significant and long-lasting perceptual impairment in all animals. In contrast, the effects we did observe were small, variable across bats, and transitory.

Congruent with the maintained behavioral performance after sound exposure, the characteristics of biosonar pulses – numbers of pulses, IPIs, and temporal patterning of IPIs – in successful flights were typically, with some exceptions, comparable on all test days. Most of the variability in pulse characteristics could be ascribed to individual differences between bats, rather than to an impact of sound exposure. Pulse numbers and IPIs are metrics for the perceptual difficulty of the task and the cognitive strategy the bat employs for solving it. Our data do not show any consistent increase in numbers of pulses after sound exposure, suggesting that the task was equally difficult in both exposure and sham conditions. Distribution of IPIs, whether presented in the form of sonar sound groups or histograms of post-IPI/pre-IPI ratios, reflect the bat’s strategy to resolve pulse- echo ambiguity^14,15,20^, which can be appreciable in the chain array task. In our experiment, IPI distributions and the complexity of sonar sound groups typically remained stable prior to and after sound exposure, indicating that the bat’s ability to resolve pulse-echo ambiguity was unaffected. Pulse characteristics sometimes varied over the three test days in the sham condition even when remaining stable over the three test days in the exposure condition. This result shows that bats can alter their emissions when challenged with a difficult task even in a sham testing condition where no extraneous sounds are present.

An exception to the overall trend of stable behavioral and acoustic performance is seen in the data from Bat B, the only bat who made significantly more errors, altered IPI distribution towards shorter IPIs, and increased pulse amplitude in successful flights after exposure to one stimulus. The basis for the different performance of this one bat is unclear. Age is a possibility; bat B may be older than the other bats, but because all bats were wild-caught, their ages are unknown. Another possibility is that aspects of the experimental situation - the bat’s individual experience with the task, its spatial memory of the array^24^, or its daily motivation to perform the task – are more important than prior sound exposure in influencing performance.

We propose two interpretations of our results. One is that sound exposure affected the bat’s individual cognitive strategy for performing the task without altering its hearing sensitivity. This interpretation is supported by the changes in IPI distributions seen in some bats after exposures. The bat’s individual criterion for rejecting pulse-echo ambiguity could alter in an attempt to solve the task, even if its hearing sensitivity is maintained. Alternatively, sound exposure might have affected a bat’s individual motivation to perform, apart from any change in hearing sensitivity or cognitive strategy. Both of these interpretations are consistent with the individual differences in performance that we observed, and with the overall pattern of results suggesting that bats are not as susceptible to TTS as are other terrestrial mammals.

## Materials and Methods

### Animals

Four wild-caught, adult big brown bats (*Eptesicus fuscus*; two males, Bats A and B; two females, Bats C and D) participated in flight experiments. Because the numbers of bats that can be captured yearly are strictly limited by Rhode Island state regulations (population monitoring is in progress to counteract effects of habitat destruction and disease), only four bats were available for testing. Bats were socially housed in a temperature- and humidity-controlled colony room (22–24 °C and 40–60% relative humidity) on a reverse (12 hr dark, 12 hr light) daily cycle. They had unlimited access to water, and received their daily food allotment (live mealworms fortified with vitamins) during experiments as rewards for successful flights. All methods were carried out in accordance with relevant federal guidelines and regulations. All experimental protocols were approved by the Brown University Institutional Animal Care and Use Committee.

### Procedure and design

Flights took place in a custom-built flight room (8.3 m long, 4.3 m wide, 2.7 m high) insulated acoustically and electrically from external ambient noise. The walls and ceiling were lined with sound-absorbent foam (SONEX) and the floor was covered to attenuate echoes. Black plastic chains (link size 4.0 cm wide, 7.5 cm long, 1.0 cm thick) were suspended from foam-covered crossbars in the ceiling straight down to the floor, and arranged in rows and columns 20 cm apart within a space measuring 600 cm long and 250 cm wide. A straight corridor 40 cm wide in the middle of this chain array was designated the flight corridor (Fig. 1). This array is denser than that used in our earlier flight experiments^13^ and features a straight rather than a curved corridor. This increased density was chosen to challenge the bats, and the new flight path was used to avoid the possibility that the three bats tested previously maintained a spatial memory of that array^24^. Flights through 40 cm corridors, whether curved or straight, are challenging for these bats^13,20^; the bat’s wingspan ranges between 30–32 cm^25^, only leaving 4–5 cm of space on either side of the animal in the corridor. Because flights took place during the bats’ subjective night, the flight room was illuminated by infrared LEDs mounted on the walls except for one dim light (90 lx) positioned on the ceiling at the release point to aid the experimenter in initiating launches.

Two Knowles SMG-0291 electret microphones recorded the bats’ biosonar pulses as they navigated the corridor. Microphones were mounted on custom-built preamplifier and high-pass filter boards and fitted with a 20-cm square foam baffle to minimize backscatter. One microphone was located on the landing wall 1.5 m above the floor and directly in line with the midpoint of the corridor crosswise, and the other was mounted on the ceiling directly above the midpoint of the corridor lengthwise. The output of both microphones was recorded on a Tascam digital recorder (Model HD-P2) at a sampling rate of 192 kHz and saved as stereo.wav files. Two video monitors, one at the release point and one next to the ceiling microphone, were used to monitor the bat’s flight path.

Bats were trained (5–7 days) until they could fly down the corridor without striking any chains and then land on the back wall. Three bats (Bats A, B, D) participated in previous experiments in which they flew through chain arrays with different chain densities, configurations, and curvatures^13,20^ while the fourth bat (Bat C) was trained specifically for this experiment. Bats were flown in the corridor for six days, three consecutive test days for each condition: Test day 1 was the baseline [24 hr pre-exposure (E1) or pre-sham (S1) day], test day 2 was the 2 min post-exposure (E2) or post-sham (S2) day, and test day 3 was the 24 hr post-exposure (E3) or post-sham (S2) day. Five to seven days later, bats received the other exposure condition; order of conditions is provided in Table 1. Bats remained in the colony room during this 5–7 day interval and did not participate in any other experiments. On each test day, experimenter #1 released the bat by hand through an elliptical opening (38 cm wide by 32 cm tall) in a gray felt curtain suspended from the ceiling of the flight room, 60 cm in front of the corridor entrance. Microphone recordings were manually started by experimenter #2 at the time of the bat’s release and manually stopped by experimenter #2 when the bat landed. Experimenter #3 retrieved the bat from its landing position and administered a food reward for a successful flight. On each test day, bats were flown for a criterion of 10–12 successful flights, with the maximum number constrained by an individual’s daily food allotment (for example, an allotment of 12 mealworms would allow a maximum of 12 successful flights; fewer if the bat dropped any rewards). An unsuccessful flight (error) was defined as not launching from experimenter #1’s hand, striking or landing on a chain, or landing on the floor of the corridor before reaching the back wall. Rewards were not given for unsuccessful flights.

### Sound exposure

Four band-limited sounds were used for exposures. Sound exposure stimulus 1 had a BW of 25 kHz (frequency range 25–50 kHz, flat amplitude); stimulus 2 had a BW of 10 kHz (frequency range 15–25 kHz, flat amplitude); stimulus 3 had a BW of 10 kHz (frequency range 20–10 kHz, downward-sweeping FM); and stimulus 4 had a BW of 5 kHz (frequency range 5–10 kHz, flat amplitude). Peak spectral levels are within the range of 95–105 dB SPL/Hz (Fig. 2). BWs of these exposure sounds were chosen to lie within the frequency range of big brown bat social calls (5–25 kHz)^21^ or echolocation pulses (FM1: 50–25 kHz)^7^. Frequencies below those used in biosonar pulses were used to assess the possibility that impairments would be observed to frequencies above the center frequency of the exposure stimulus, as demonstrated in other terrestrial mammals (the half-octave shift^23^). Thus, exposure to stimuli 2, 3, or 4 might produce a decline in hearing sensitivity to higher frequencies within FM1 of the bat’s own pulses. If thresholds to either emitted pulses or returning echoes are increased by sound exposure, then this would result in impaired performance.

Broadband noise was generated by an Elgenco analog random noise generator and band-pass filtered (Rockland 422 dual Hi/Low filter, 24 dB/octave roll-off) to the appropriate ranges to create the three band-limited, flat-amplitude sounds. FM sweeps were digitized by Adobe Audition (sampling rate 96 kHz) and band-pass filtered from 10–20 kHz. Stimuli were amplified (Harmon-Kardon P645 power amplifier) and broadcast through an ultrasonic leaf tweeter (Panasonic EAS 10TH) located 8 cm away from the nearest wall of the 15 cm^3^ mesh cage holding the bat during sound or sham exposures. The tweeter and mesh cage were placed in one corner of the flight room, outside the chain array. Sound pressure levels at different positions in the mesh cage were measured using a Brüel & Kjaer model 4135 (¼ in) condenser microphone. The mean level of each stimulus was adjusted to be 123 dB SPL rms (sound exposure level, SEL, of 158 dB) at the center of the cage, but varied between 119–124 dB SPL at various positions in the mesh cage. Sound and sham exposures lasted 1 hr. For sham exposures, the bats were placed into the mesh cage but the power amplifier was turned off. During both sound and sham exposures, bats were free to move within the cage, but they typically adopted a position hanging on one of its sides, and remained stationary throughout the 1 hr exposure/sham. At the end of this 1 hr period, the bat was immediately taken from the mesh cage to the release position at the entrance of the flight corridor, and 2 min later was released to begin the series of flights.

### Data analysis

Because numbers of pulses and IPIs were identical at the wall and ceiling microphones, only data from the wall microphone were analyzed. Recordings were high-pass filtered at 15 kHz to reduce ambient noise and analyzed using custom-written MATLAB (R2014a) scripts. The audio files for each flight typically varied around 3 s, because the recording device was manually switched on at the time of release and switched off at the time of landing. For analysis of successful flights, these recordings were trimmed to a standard time interval of 1.5 s so as to include only those pulses the bat emitted during sustained flight within the corridor, and to eliminate any variability produced by the manual onset/offset of the recording device. The demarcation of the 1.5 s time interval in each audio file was made manually backwards from the beginning of the landing buzz, a sequence of low amplitude, high repetition rate pulses made by bats when landing on a surface (Fig. 4a); in practice, this resulted in analysis intervals of 1.43 to 1.55 s (the range exists so as to not to eliminate any pulses within sonar sound groups that occurred at the beginning of the flight). Unsuccessful flights were typically shorter than successful flights, because in those cases the bat did not reach the back wall. Those flights were truncated manually backwards from the time at which the bat landed on a chain or on the floor, as indicated by a landing-type buzz (Fig. 4c), to the estimated time at which the bat entered the corridor, as marked by a decrease in IPI from the long IPI pulses (90–100 ms; Fig. 4d) emitted when launching and before entering the corridor. A custom-written MATLAB script calculated numbers of pulses and IPIs. The distribution of IPIs was plotted as the relationship between post-IPI and pre-IPI intervals (that is, the interval between each subsequent pulse compared to each preceding pulse; Fig. 4b). Sonar sound groups were identified using a modified version of published criteria^19^. IPIs less than 12 ms were eliminated, as these likely are produced by strong echoes from the chains or the floor.

Statistical differences in numbers of successful and unsuccessful flights for each stimulus were analyzed by fitting a Bayesian binomial generalized linear model with an uninformative Cauchy prior to obtain the Bayesian odds-ratio^26^. A Bayesian analysis allows detection of ‘invariances,’ i.e., detecting when phenomena (in this case, flight performance) do not change even in a salient context (in this case, intense sound exposure)^22^. Statistical differences in the numbers of pulses and in IPIs between sound and sham exposures for each stimulus were assessed using RM-ANOVA, with Test Day as the repeated measure and Bat as the between-subjects measure, when appropriate. Violations from sphericity were corrected using the Greenhouse-Geisser procedure. *P* values for paired comparisons on the overall ANOVA were adjusted using the Bonferroni criterion. Two-sample Kolmogorov-Smirnov (K-S) tests were used to compare changes in the distributions of IPIs across test days.

## Electronic supplementary material

Supplementary figure 1

## Data Availability

Data are deposited in the Brown University data archive 10.7301/Z04B2ZSD.
