# Supplementary material for: Big brown bats (Eptesicus fuscus) successfully navigate through clutter after exposure to intense band-limited sound
Source: Sci Rep. 2018 Sep 10;8:13555. doi: 10.1038/s41598-018-31872-x (PMC6131230; doi:10.1038/s41598-018-31872-x)
Supplement: Supplementary file 1 — Supplementary figure 1 [file 41598_2018_31872_MOESM1_ESM.pdf]

Supplementary information for

Big brown bats (*Eptesicus fuscus*) successfully navigate through clutter after exposure to intense band-limited sound

Andrea Megela Simmons, Alexandra Ertman, Kelsey N. Hom, & James A. Simmons

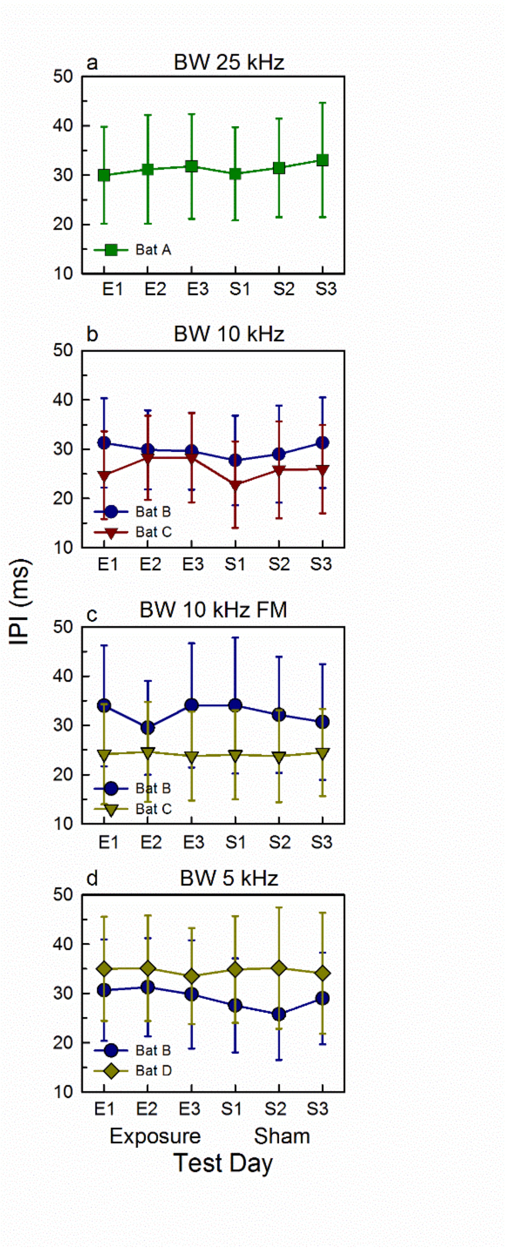

Supplemental Figure 1. Mean IPI does not vary consistently after sound exposure. In each plot, data points are means +/- 1 standard deviation in the first 10 successful flights on each test day (x axis), with individual bats identified by a unique color and symbol. **a**: BW 25 kHz; **b**: BW 10 kHz; **c**: BW 10 kHz FM; **d**: BW 5 kHz. Over the entire dataset, there is no statistical decrease in IPI on test day E2 compared to test days E1 or S2, as would be predicted by a hypothesis of perceptual impairment.
